# Prediction of electroconvulsive therapy outcome: A network analysis approach

**DOI:** 10.1111/acps.13770

**Published:** 2024-11-11

**Authors:** Tessa F. Blanken, Rob Kok, Jasmien Obbels, Simon Lambrichts, Pascal Sienaert, Esmée Verwijk

**Affiliations:** ^1^ Department of Psychology University of Amsterdam Amsterdam Netherlands; ^2^ Department of Old Age Psychiatry Parnassia Psychiatric Institute The Hague Netherlands; ^3^ Department of Neurosciences, University Psychiatric Center KU Leuven, Research Group Psychiatry Academic Center for ECT and Neuromodulation (AcCENT) Leuven Belgium; ^4^ KU Leuven University Psychiatric Center KU Leuven Leuven Belgium; ^5^ Department of Medical Psychology Amsterdam UMC Amsterdam Netherlands; ^6^ ECT department Parnassia Psychiatric Institute The Hague Netherlands

**Keywords:** depression, electroconvulsive therapy, network analysis, prediction

## Abstract

**Objective:**

While electroconvulsive therapy (ECT) for the treatment of major depressive disorder is effective, individual response is variable and difficult to predict. These difficulties may in part result from heterogeneity at the symptom level. We aim to predict remission using baseline depression symptoms, taking the associations among symptoms into account, by using a network analysis approach.

**Method:**

We combined individual patient data from two randomized controlled trials (total *N* = 161) and estimated a Mixed Graphical Model to estimate which baseline depression symptoms (corresponding to HRSD‐17 items) uniquely predicted remission (defined as either HRSD≤7 or MADRS<10). We included study as moderator to evaluate study heterogeneity. For symptoms directly predictive of remission we computed odds ratios.

**Results:**

Three baseline symptoms were uniquely predictive of remission: suicidality negatively predicted remission (OR = 0.75; bootstrapped confidence interval (bCI) = 0.44–1.00) whereas retardation (OR = 1.21; bCI = 1.00–2.02) and hypochondriasis (OR = 1.31; bCI = 1.00–2.25) positively predicted remission. The estimated effects did not differ across trials as no moderation effects were found.

**Conclusion:**

By using a network analysis approach this study identified that the presence of suicidal ideation predicts an overall worse treatment outcome. Psychomotor retardation and hypochondriasis, on the other hand, seem to be associated with a better outcome.

**Significant outcomes**

- Three baseline depression symptoms were directly and uniquely predictive of remission: ‘retardation’ and ‘hypochondriasis’ both positively predicted remission, whereas ‘suicidality’ was a negative predictor of remission.

**Limitations**

- The use of the HRSD‐17 to assess individual depressive symptoms limits the generalizability of our findings.
- Suicidality was measured with a single item from the HDRS‐17.
- The current network estimation technique does not adequately capture the ordinal structure in the data, which may bias the estimates.

## 1 INTRODUCTION

Although the overall efficacy of electroconvulsive therapy (ECT) for the treatment of major depressive disorder (MDD) is well established, the individual response to ECT is variable and difficult to predict.^1^ So far, outcome predictors include older age, presence of psychotic and psychomotor symptoms, absence of personality disorder and a shorter episode duration.^2^, ^3^, ^4^ Analyses of melancholic features and depression severity are inconclusive due to heterogeneity between studies.^3^ Reliable prediction of treatment success may lead to a more adequate indication for ECT.

Most previous studies that investigated the association between depression severity and treatment effect looked at the overall severity of depressive symptoms using sum scores. This would assume that depression consists of a single underlying construct where all symptoms are interchangeable and equally good indicators of the underlying depression.^5^ However, there is increasing evidence that depression has a multidimensional character where there is a high degree of variability in symptoms between and within patients.^6^ For example, individual depressive symptoms have different biological underpinnings,^7^ differently impact psychosocial functioning^8^ and have different risk factors.^9^ It may therefore be important to investigate the relative contributions of depressive symptoms in finding clinical predictors for treatment effect.

More recently, network analysis has been used to identify specific symptoms that are targeted throughout treatment.^10^, ^11^ For example, in depression treatment cognitive behavioral therapy versus antidepressant medication targets different depression symptoms.^11^ Interestingly, in different studies it was shown that investigating treatment effects at the symptom level may reveal differential pathways targeted by treatment that were obscured when comparing the treatments at the overall severity level.^12^, ^13^ These findings highlight the potential and importance of investigating predictive effects not only at the overall severity level, but also at the symptom level. In addition, these studies show that symptoms may play a differential role in the network and may therefore also contribute differently to predicting clinical treatment outcomes.

In the current study we aimed, for the first time, to investigate whether there are specific depressive symptoms at baseline that may predict ECT treatment response. Given that depressive symptoms are themselves related, we used network analysis to identify unique predictive effects: symptoms that predict ECT treatment response while taking the other depressive symptoms and their associations into account.^14^ Identifying symptoms that may uniquely and directly predict response to ECT treatment can better identify patients who will benefit or not.

### 1.1 Aims of the study

We present the results of a network analysis aimed to identify unique predictive effects of depressive symptoms: which baseline depressive symptoms predict ECT treatment outcome while taking the other depressive symptoms and their associations into account.

## 2 METHODS

### 2.1 Study sample

In the current study we re‐analyzed data from two randomized clinical trials (RCTs) comparing different ECT techniques for major depression.^15^, ^16^ Both RCTs included patients older than 18 who met the DSM‐IV criteria for a depressive episode, either unipolar or bipolar. Patients with a history of schizophrenia were excluded. In addition, Sienaert et al.^15^ excluded patients with neurological illness, cognitive disorder, substance abuse or dependence within the previous year, or ECT within the past 6 months; and Spaans et al.^16^ also excluded patients with schizoaffective disorder or dementia. For the current re‐analyses, we included all patients for whom a treatment outcome variable was available. From the total randomized group of *n* = 197 patients, this resulted in *n* = 161 patients (*n* = 81 patients reported in Sienaert et al.^15^ and *n* = 80 reported in Spaans et al.^16^). The demographic and clinical parameters were comparable between the included group of patients and those with missing outcomes (*n* = 36) (data not shown).

### 2.2 Treatment

All patients were treated with ECT twice a week until remission was achieved or there was a plateau in improvement over at least two consecutive evaluations. The patients received bifrontal (BF) or right unilateral (RUL) ECT using ultra‐brief pulse (UBP) or brief pulse (BP) width by random assignment.

In the study of Sienaert et al.^15^ remission was defined as a Hamilton Rating Scale for Depression^17^ (HRSD) score (17‐item HRSD) of ≤7, which corresponds to full remission.^18^ Spaans et al.^16^ defined remission during the RCT phase as a Montgomery‐Åsberg Depression Rating Scale^19^ (MADRS) score < 10 on two consecutive measurements. More detailed information on the methodology and results of these RCTs are published elsewhere.^15^, ^16^

### 2.3 Measures

In both studies, the 17‐item HRSD was used to assess depressive symptom severity at baseline. The HRSD‐17 is a clinician‐administered depression assessment scale pertaining to depression symptoms over the past week. Most items are scored on a scale from 0 to 4, except for items on somatic and genital symptoms, weight loss, and insight (0–2).

### 2.4 Statistical Analysis

Estimation. To investigate which baseline depression symptoms were related to remission, we used Network Outcome Analysis (NOA^14^) by estimating a mixed graphical model^20^ including all 17 HRSD items representing the depressive symptoms as well as a binary variable indicating whether someone remitted or not (0: no, 1: yes). Despite being measured on an ordinal scale, all symptoms were included as continuous variables as we had too few observations to include them as categorial. The binary outcome variable was included as categorical. Given the low sample‐size relative to the number of estimated parameters, we used L1‐regularized regression (LASSO) to prevent the inclusion of spurious edges due to sampling variation.^21^, ^22^

By estimating the network using LASSO regularization, we aim to select a model that generalizes well to new samples. Crucial in using LASSO regularization is selecting a regularization parameter lambda. Selecting between different regularization parameters represents selecting between different sets of predictors being included in the regression model.* The regularization parameter thus essentially determines which edges are included in our network. There are different approaches to select the regularization parameter, and in our paper we used 10‐fold cross validation, where the out‐of‐fold prediction errors are approximating the out‐of‐sample prediction errors we would obtain in new samples from the same population. We therefore set parameters to zero that do not sufficiently improve out‐of‐fold predictions. Since our sample size is finite, this means that we likely will incorrectly set some (smaller) parameters to zero that are nonzero in the population. The probability of this happening depends on the overall model and specifically the size of these parameters. However, we can look at the smallest parameter that was included in the model and can conclude that we did not incorrectly set parameters to zero that were smaller than this parameter.

It is important to note that selecting a regularization parameter replaces the model selection done with hypothesis testing in more traditional approaches (null hypothesis significance testing; NHST). Consequently, when estimating a network using LASSO regularization we do not obtain significance levels of the edges included in the network. This also applies to any inferences that are based on the estimated edge weights, such as the computed odds ratios (see inferences below), as these are a transformation of the edge weights using the exponential function. Even though network estimation using LASSO regularization does not provide us with significance levels, we can still compute sampling distributions for each parameter using resampling techniques. We computed 500 bootstrap samples to get a sense of the variance in our estimates,^23^ see supplement for more information. However, in interpreting these bootstrapped sampling distributions note that the sampling distributions are biased because of the regularization. Regularization shrinks the parameters towards zero, allowing us to select which edges to include in the network by setting some parameters exactly to zero. Since this regularization (or bias) affects all parameters as well as their sampling distributions, the sampling distributions cannot be interpreted as regular confidence intervals in terms of coverage (i.e., to have the property to contain the true parameter value with probability 1‐ɑ).^24^, ^25^ We therefore refer to the reported uncertainty estimates as bootstrapped confidence intervals (bCI).

In conclusion, comparing model selection using LASSO regularization to NHST there are two important differences to keep in mind. First, LASSO regularization does not give significance levels of the included edges. To nonetheless get an indication of the variation in the estimated parameters, we computed bootstrapped sampling distributions. Be aware, however, that these cannot be interpreted as regular confidence intervals given the biased estimates. Second, in both scenarios (LASSO vs. NHST) we may set effects to zero that were actually nonzero in the populations (i.e., false positives). However, with LASSO regularization we have an idea of how small the missed effects are by looking at the smallest included parameter.

For the estimated network, we computed the predictability of each symptom, representing the proportion of variance explained by all the other symptoms in the network. Finally, to maximize the power to identify predictive effects, we estimated the network on both samples combined. To evaluate study heterogeneity, we estimated a moderated network model^26^ to evaluate whether ‘study’ moderated any of the relationships in the network.

Interpretation. We visualized the estimated mixed graphical model as a network, in which all variables are included as nodes (depressive symptoms as circles, the remission outcome variable as a square) that are connected by links that represent the conditional dependence relations (blue links represent positive, and red links represent negative conditional dependence relations). This means that a link between two nodes represents the unique association between those two variables, while controlling for all the other variables in the network. Correspondingly, any link between a depressive symptom and the remission outcome variable thus indicates a unique, direct predictive effect of that depression symptom at baseline for eventual remission, while controlling for the other depressive symptoms. In interpreting these links, keep in mind that the links are conditioned on the rest of the network, which can be thought of as analogue to the effect of a single symptom in two patients who, apart from this symptom, show the same overall severity. Be aware, however, that we have estimated one network on the entire group, so the estimated effects cannot be immediately generalized to single patients but show general patterns in this population of patients.

Even though the mixed graphical model estimates bidirectional links, since the assessment of baseline depressive symptoms precedes eventual remission, the symptoms can predict remission, but not vice versa. Thus, any depressive symptom that is linked to remission uniquely and directly predicts remission by ECT treatment. The predictability of each symptom is shown in a ring around the node: a completely filled ring indicates that 100% of the variance is explained, while an empty ring corresponds to a proportion of explained variance of 0%.^27^

Evaluation. To evaluate any estimated links between baseline depressive symptoms and remission, we computed the odds ratios of these conditional dependence relations. That is, for each baseline depressive symptom that is directly associated to remission, we computed the odds of attaining remission relative to not attaining remission.

All analyses were performed in R (version: 4.3.1) with packages mgm (version: 1.2–14) and qgraph (version: 1.9.5). R‐scripts are available in an OSF repository (https://osf.io/43f8r/).

## 3 RESULTS

Table 1 shows the main demographical and clinical characteristics of our study group. Of the 161 patients that received ECT, 100 (62.1%) remitted, and they did not differ significantly from patients who did not remit (Table 1).

**TABLE 1:** Main demographical and clinical characteristics (mean ± SD) for the complete sample (*N* = 161) as well as for remitters (*n* = 100) and non‐remitters (*n* = 61) separately.

	Complete sample *N* = 161	Remitters *n* = 100 (62.1%)	Non‐remitters *n* = 61 (37.9%)	Difference remitters and non‐remitters
Age, years (*n* = 158)	58.0 ± 14.1	59.4 ± 14.0	55.7 ± 14.1	*t*(124.53) = −1.62, *p* = 0.11
Female (*n* = 159)	103 (64.8%)	68 / 99 (68.7%)	35 / 60 (58.3%)	χ(1) = 1.33, *p* = 0.25
Number of ECT sessions (*n* = 152)	11.0 ± 3.8	10.9 ± 2.0	11.2 ± 4.5	*t*(146.10) = 0.54, *p* = 0.59
Bipolar depression	27 (16.8%)	18 (18.0%)	9 (14.8%)	χ(1) = 0.10, *p* = 0.75
Baseline HRSD score	25.8 ± 7.5	25.9 ± 8.0	25.6 ± 6.7	*t*(143.42) = −0.20, *p* = 0.84
End‐point HRSD score (*n* = 156)	11.4 ± 7.7	6.9 ± 3.9	18.8 ± 6.7	*t*(79.91) = 12.2, *p* < 0.001

**FIGURE 1:**
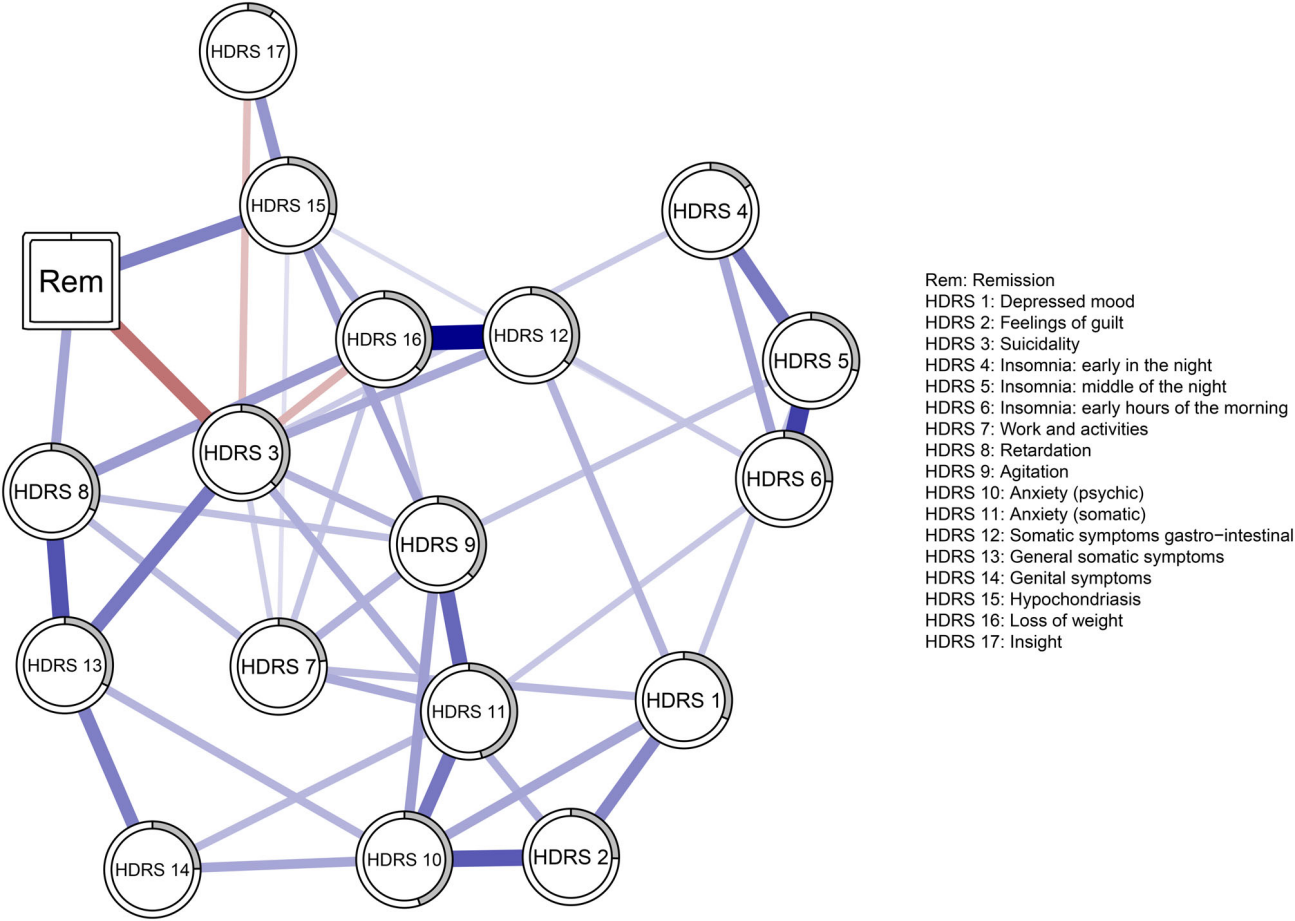
Network visualization of the regularized mixed graphical model. The network includes the HDRS items (circles) and the remission outcome variable (square). The links represent conditional dependence relations: The unique association between two variables after controlling for all the other variables in the network. Blue links represent positive relations, and red links negative relations. The proportion of explained variance in each HDRS item by the other variables in the network is shown in a ring around the node, where a completely filled ring corresponds to 100% and a completely empty ring to 0% explained variance.

NOA identified three baseline depressive symptoms that were directly and uniquely predictive of remission (Figure 1): ‘retardation’ and ‘hypochondriasis’ both positively predicted remission, whereas ‘suicidality’ was a negative predictor of remission. Conditioning on the rest of the network–analogous to seeing two patients with the same HDRS total score–this indicates that patients with a symptom profile including more severe ‘retardation’ and ‘hypochondriasis’ are more likely to remit than patients with different symptom profiles. Conversely, conditioning on the rest of the network, patients who score higher on suicidality are less likely to remit than patients with less severe or no suicidality.

The negative link between suicidality and remission corresponds to an odds ratio of 0.75 (bCI = 0.44–1.00), indicating that with every 1‐point increase for the suicidality item, the odds of remission decreased by 25%. Compared with a suicidality score of 0 (no suicidality), a suicidality score of 4 (indicating ‘attempt at suicide’) thus corresponds to being 3.2 times less likely to remit. The positive link between retardation and remission indicates that with every 1‐point increase for the retardation item, the odds of remission increased by 21% (OR = 1.21; bCI = 1.00–2.02). A retardation score of 4 (‘complete stupor’) corresponds to being 2.1 times more likely to benefit from ECT and remit than a score of 0 (indicating ‘normal speech and thought’). Finally, the positive link between hypochondriasis and remission indicates that for every 1‐point increase for the hypochondriasis item, the odds of remission increased by 31% (OR = 1.31; bCI = 1.00–2.25). Thus, compared with no hypochondriasis (item score of 0) a hypochondriasis score of 4 (indicating ‘hypochondriacal delusions’) corresponds to being 3.0 times more likely to remit.

The moderation analyses showed that none of the identified relations between the baseline symptoms ‘suicidality’, ‘retardation’, and ‘hypochondriasis’ was moderated by ‘study’. Although we cannot interpret the absence of an effect as proof that there are no differences between the studies (“absence of evidence” is not “evidence of absence”), it suggests that the unique predictive effects of ‘suicidality, ‘retardation,’ and ‘hypochondriasis' are likely to be robust across the two RCTs.

To further assess the stability of the estimated networks we conducted bootstrap analyses. These stability analyses indicated that the links between remission and the three symptoms were quite robust: the link with ‘suicidality, ‘retardation’, and ‘hypochondriasis' was retrieved in 83%, 84%, and 79% of the bootstrapped samples, respectively.

## 4 DISCUSSION

This study identified three depressive symptoms at baseline that were directly and uniquely predictive of remission in patients with depression treated with ECT: ‘retardation,’ ‘hypochondriasis,’ and ‘suicidality’. Psychomotor retardation and hypochondriasis both positively predicted remission, indicating that patients with a symptom profile including higher scores on these symptoms are more likely to remit than patients with different symptom profiles. In contrast, suicidal ideation was a negative predictor of remission, which indicates that patients with more severe suicidality are less likely to remit than patients without suicidal thoughts.

We for the first time used network analysis to investigate the unique predictive effects of individual baseline depressive symptoms of ECT treatment outcome. In contrast to more traditional approaches, such as predicting treatment outcome by baseline symptoms using regression analyses,^28^, ^29^ network analysis has the advantage of taking the interdependencies between the symptoms into account. As becomes clear from past literature^30^ and the current study (see Figure 1), the depressive symptoms are themselves strongly correlated. Taking these interdependencies into account allows us to differentiate between direct and indirect predictive effects.^14^ Clinically, distinguishing symptoms that directly predict treatment outcome (e.g., hypochondriasis) from symptoms that do so indirectly through their association with directly related symptoms (e.g., agitation) may help identify key predictors that contain most information on treatment outcomes.

Our finding that psychomotor retardation and hypchondriasis are associated with a better treatment outcome in depression provides additional confirmation to a growing body of literature.^2^, ^3^ However, much less is known in the literature about suicidality and the effect on ECT outcome. Our analysis showed that this symptom is a predictor for a worse overall ECT outcome. The impact of baseline suicidality on the outcome of ECT was recently reported by Sienaert et al.^31^ In a register‐study they found suicidal ideation at baseline to be associated with lower ECT response and remission rates. Nevertheless, suicidal ideation was reduced during the course of treatment in a large proportion of their patients, which is something that is more broadly studied and confirmed.^32^, ^33^

Suicidality is not only a negative predictor for ECT treatment outcome, but also for other depression treatments such as medication and cognitive behavioral therapy.^34^, ^35^ In the treatment of depression, we must thus acknowledge the fact that patients who are suicidal before treatment, might recover less well from their depression. Nonetheless, ECT seems superior within the treatment arsenal in treating suicidality.^32^, ^36^ There is growing evidence for an acute anti‐suicidal effect of (es)ketamine as well.^37^ Whether baseline suicidality is associated with an overall worse treatment outcome remains to be studied.

The use of the HRSD‐17 to assess individual depressive symptoms limits the generalizability of our findings. Selection of a particular scale may bias results as different instruments have been shown to capture different aspects of the heterogeneous depressive syndrome. In fact, there is a considerable difference in item content across instruments.^38^

In addition, looking at the symptom profiles as we did in our study, another limitation is that suicidality was measured with a single item from the HDRS‐17. In future research, valid measures such as the Depressive Symptom Index Suicidality Subscale, the Suicidal Ideation Attributes Scale (SIDAS) or Columbia–Suicide Severity Rating Scale (C‐SSRS) can be used.^39^, ^40^ However, despite using only one item, our current study complements the recent findings by Sienaert et al.^31^ as the suicidal ideation was reported by clinicians instead of self‐rated and our studies were prospectively collected within two RCTs.

One downside of network analysis is that many relationships are estimated and particularly in RCTs, the ratio of number of observations relative to the number of estimated links is small. Therefore, we have used regularization, a technique developed in machine learning and particularly well suited in cases where the number of observations relative to the number of parameters is low.^22^ We further assessed the stability of our estimated links and the three predictive links in our estimated network were robust across bootstrap samples. However, replications of these findings in independent datasets would be required. In addition, it would be interesting to explore in a larger dataset whether factors such as age, sex, or episode duration may influence the established relations by including these in the network as well. Another future recommendation is to also apply the network analysis method to unravel the network of predictive factors that lead to cognitive side effects after ECT.

Finally, in our current network estimation we included all variables as continuous variables, despite their assessment on an ordinal scale. While there is no large imbalance in the response categories that are observed in the data (see Figure S2), including all variables as continuous remains an important limitation. Unfortunately, within the current frequentist statistical framework there is no statistical model available for ordinal Likert scale type data. The only other opportunity would be to binarize the data. However, how to binarize the data is not trivial and may lead to a loss of information. Therefore, we are currently limited in the frequentist network modeling of ordinal data, which remains a limitation of the current analyses.

In conclusion, clinicians should be aware that the presence of suicidal ideation might be associated with an overall poorer depression treatment outcome, although ECT can reduce suicidal ideation. Psychomotor symptoms and hypochondriasis, on the other hand, seem to be associated with a better treatment outcome.

## CONFLICT OF INTEREST STATEMENT

The authors declare no conflicts of interest.

### PEER REVIEW

The peer review history for this article is available at https://www.webofscience.com/api/gateway/wos/peer‐review/10.1111/acps.13770.

## Supporting information


**Data S1.** Supporting Information.

## Data Availability

The data that support the findings of this study are available on request from the corresponding author. The data are not publicly available due to privacy or ethical restrictions.

## Footnotes

* Technically, in L1‐regularized regression we minimize the negative log likelihood of the data together with a penalty term. The penalty term is the sum of the absolute value of all slope parameters (not the intercepts). The relative weight of the negative log likelihood of the data and the penalty term is determined by the regularization parameter lambda. The regularization determines the amount of shrinkage (bias) of the parameters towards zero, and thus how many parameters are put exactly to zero (i.e., selection of parameters).
