# Supplementary material for: Prediction of electroconvulsive therapy outcome: A network analysis approach
Source: Acta Psychiatr Scand. 2024 Nov 11;151(4):521–8. doi: 10.1111/acps.13770 (PMC11884910; doi:10.1111/acps.13770)

**Supplementary Material**

*Stability analyses*

To evaluate the accuracy of the estimated edges we created five hundred bootstrap samples and fitted the model in each of them. This procedure gives us five hundred estimates of each edge, from which we can obtain a sampling distribution, of which the 5% and 95% quantiles are plotted in Figure S1. In interpreting the sampling distributions, please note that because we used LASSO regularisation in estimating the network that the sampling distribution of the edge weights is biased towards zero. Accordingly, the 5% and 95% quantiles should not be interpreted as confidence intervals, as they can include zero even though the confidence intervals would not include zero. Next to the 5% and 95% quantiles of the estimated edge weights, the plot also shows the proportion of bootstrap samples that included the edge in the network. For the links of interest that involve remission, it can be seen that these are included in 84% of the bootstrap samples for ‘hypochondriasis’ (Rem – Ham 15), in 79% of the bootstrap samples for ‘retardation’ (Rem – Ham 8), and in 83% of the samples for ‘suicide’ (Rem – Ham 3). For further details, see Epskamp, Borsboom, & Fried (2017).

 **Figure S1.** Bootstrapped sampling distributions.

**Figure S2.** Marginal distributions of HDRS items split by response category.


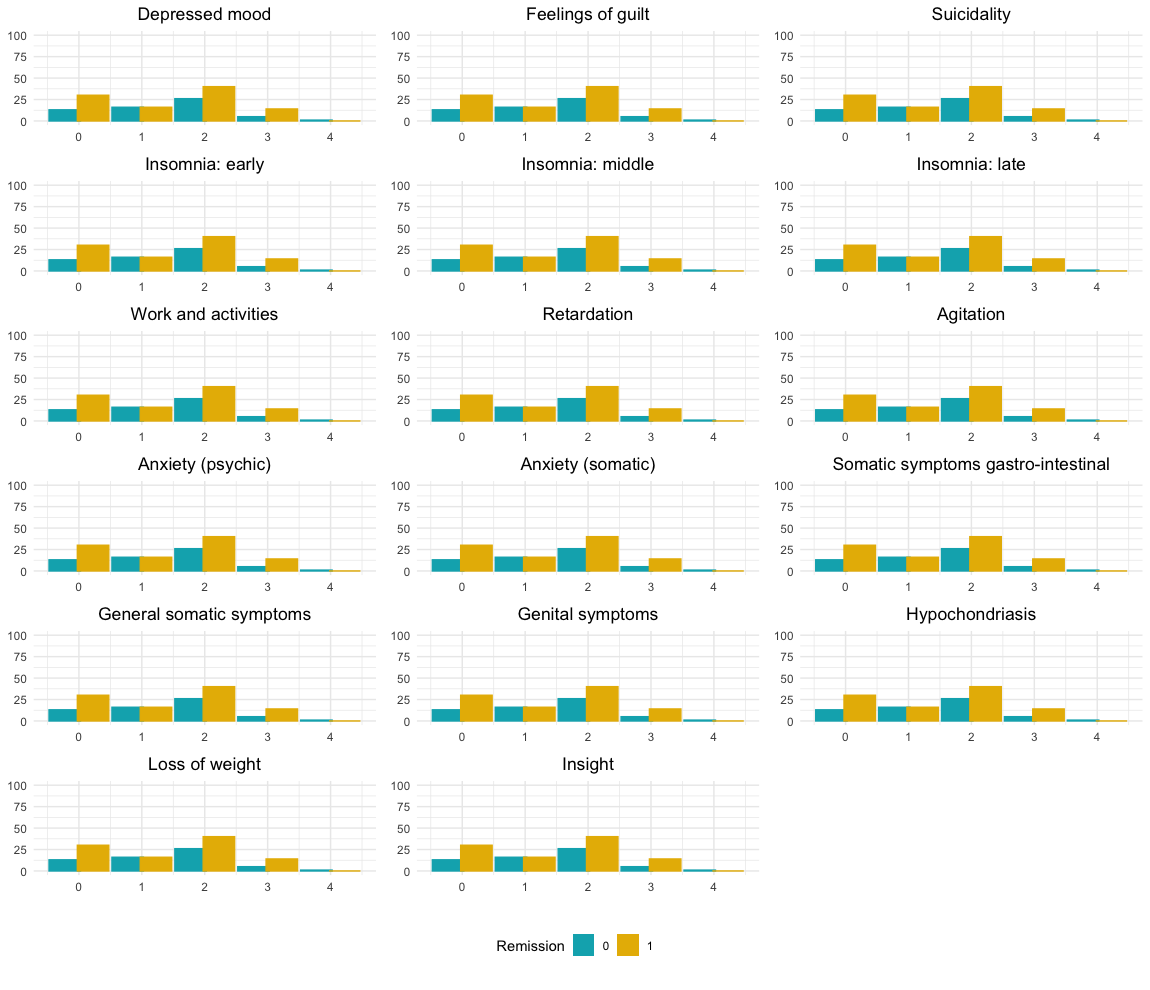

Supplement: Supplementary file 1 — Data S1. Supporting Information. [file ACPS-151-521-s001.docx]
